# Cross-level analysis of molecular and neurobehavioral function in a prospective series of patients with germline heterozygous *PTEN* mutations with and without autism

**DOI:** 10.1186/s13229-020-00406-6

**Published:** 2021-01-28

**Authors:** Thomas W. Frazier, Ritika Jaini, Robyn M. Busch, Matthew Wolf, Tammy Sadler, Patricia Klaas, Antonio Y. Hardan, Julian A. Martinez-Agosto, Mustafa Sahin, Charis Eng, the Developmental Synaptopathies Consortium, Simon K. Warfield, Benoit Scherrer, Kira Dies, Rajna Filip-Dhima, Amanda Gulsrud, Ellen Hanson, Jennifer M. Phillips

**Affiliations:** 1 Department of Psychology, John Carroll University, University Heights, OH 44118 USA; 2 Autism Speaks, Cleveland, OH USA; 3 Genomic Medicine Institute, Lerner Research Institute, Cleveland Clinic, Cleveland, OH 44195 USA; 4 Department of Neurology and Epilepsy Center, Neurological Institute, Cleveland Clinic, Cleveland, OH 44195 USA; 5 Department of Child Psychiatry and Behavioral Sciences, Stanford University School of Medicine, Palo Alto, CA USA; 6 Department of Human Genetics, UCLA, Los Angeles, CA USA; 7 Translational Neurosciences Center, Department of Neurology, Boston Children’s Hospital and Harvard Medical School, Boston, MA 02115 USA; 8 Center for Personalized Genetic Healthcare, Cleveland Clinic Community Care and Population Health, Cleveland, OH 44195 USA; 9 Department of Genetics and Genome Sciences, Case Western Reserve University School of Medicine, Cleveland, OH 44106 USA; 10 Cleveland Clinic Genomic Medicine Institute, 9500 Euclid Avenue, NE-50, Cleveland, OH 44195 USA

**Keywords:** PTEN, Protein, Molecular, Autism spectrum disorder, Cognition, Behavior

## Abstract

**Background:**

*PTEN* is a well-established risk gene for autism spectrum disorder (ASD). Yet, little is known about how *PTEN* mutations and associated molecular processes influence neurobehavioral function in mutation carriers with (PTEN-ASD) and without ASD (PTEN no-ASD). The primary aim of the present study was to examine group differences in peripheral blood-derived PTEN pathway protein levels between PTEN-ASD, PTEN no-ASD, and idiopathic macrocephalic ASD patients (macro-ASD). Secondarily, associations between protein levels and neurobehavioral functions were examined in the full cohort.

**Methods:**

Patients were recruited at four tertiary medical centers. Peripheral blood-derived protein levels from canonical PTEN pathways (PI3K/AKT and MAPK/ERK) were analyzed using Western blot analyses blinded to genotype and ASD status. Neurobehavioral measures included standardized assessments of global cognitive ability and multiple neurobehavioral domains. Analysis of variance models examined group differences in demographic, neurobehavioral, and protein measures. Bivariate correlations, structural models, and statistical learning procedures estimated associations between molecular and neurobehavioral variables. To complement patient data, Western blots for downstream proteins were generated to evaluate canonical PTEN pathways in the PTEN-m3m4 mouse model.

**Results:**

Participants included 61 patients (25 PTEN-ASD, 16 PTEN no-ASD, and 20 macro-ASD). Decreased PTEN and S6 were observed in both *PTEN* mutation groups. Reductions in MnSOD and increases in P-S6 were observed in ASD groups. Elevated neural P-AKT/AKT and P-S6/S6 from PTEN murine models parallel our patient observations. Patient PTEN and AKT levels were independently associated with global cognitive ability, and p27 expression was associated with frontal sub-cortical functions. As a group, molecular measures added significant predictive value to several neurobehavioral domains over and above *PTEN* mutation status.

**Limitations:**

Sample sizes were small, precluding within-group analyses. Protein and neurobehavioral data were limited to a single evaluation. A small number of patients were excluded with invalid protein data, and cognitively impaired patients had missing data on some assessments.

**Conclusions:**

Several canonical PTEN pathway molecules appear to influence the presence of ASD and modify neurobehavioral function in *PTEN* mutation patients. Protein assays of the PTEN pathway may be useful for predicting neurobehavioral outcomes in PTEN patients. Future longitudinal analyses are needed to replicate these findings and evaluate within-group relationships between protein and neurobehavioral measures.

**Trial registration:**

ClinicalTrials.gov Identifier NCT02461446

## Background

*PTEN* encodes a dual-specificity phosphatase that inhibits PI3K/AKT/mTOR and MAPK/ERK pathway activation [1] and is an important regulator of neural connectivity and plasticity [2]. A suggestive association between germline heterozygous *PTEN* mutations and autism spectrum disorder (ASD) was first identified through cases of ASD with extreme macrocephaly [3, 4]. Subsequent small cohort studies identified an enriched number of germline *PTEN* mutations in ASD cases with macrocephaly [5–7]. Larger clinical cohort studies of ASD and/or intellectual disability with macrocephaly also found an enrichment of *PTEN* mutations [8–12], resulting in a weighted average of 7% of macrocephalic ASD and translating to approximately 1% of all ASD cases [13]. Exome and genome sequencing studies of large ASD cohorts have confirmed *PTEN* as well-established risk gene for ASD and neurodevelopmental disorders [14–20], and recent case series have suggested that 25–50% of children with *PTEN* mutations are identified with ASD [21, 22].

The association between *PTEN* and ASD has been further supported by nearly two decades of mouse model studies [23–29]. These investigations have shown that *PTEN* loss impairs brain development and leads to aberrant brain connectivity [13], a well-replicated phenotype observed in idiopathic ASD cases [30–32]. A recent knock-in model that results in cytoplasmic predominance of PTEN was found to have expression changes in genes important for white matter development, along with social and motor deficits reminiscent of ASD cases with intact cognitive ability [28, 29]. Findings from this model also suggest that the impact on neurobehavior is highly variable, consistent with observations in human patients [33–35].

Discoveries from cohorts of human patients with *PTEN* mutations have generally mirrored the *PTEN* mouse literature. For example, an initial study of patients with germline heterozygous *PTEN* mutations and ASD identified major increases in structural white matter volumes as well as hypo-intensities on structural MRI images, consistent with neuropathology observed in mouse models [33]. In this cohort, PTEN loss was associated with white matter abnormalities, which, in turn, predicted greater cognitive deficits. More recent investigations of *PTEN* mutation patients with (PTEN-ASD) and without ASD (PTEN no-ASD) have observed reductions in frontal sub-cortical neurobehavioral functions in most cases. However, impairments in general cognitive ability are only common in PTEN-ASD. For most neurobehavioral measures, variability within and across patient groups was very large, with some patients functioning in the average or above average range in most neurocognitive domains and other patients showing moderate to severe global cognitive dysfunction [33–35]. Thus, it is important to understand what molecular factors may be driving variability in neurobehavioral function to identify potential treatment targets and inform individual patient prediction.

A key initial question to address is whether PTEN-related pathway proteins show differences in expression due to the presence of a *PTEN* mutation or the presence of ASD regardless of mutation status. In our small initial cohort, PTEN-ASD cases (*n* = 17) had significantly reduced PTEN levels [33], but the magnitude of reduction was modest, suggesting that the healthy allele may be partially compensating for the deleterious allele. Furthermore, it is unclear whether reductions in PTEN are specific to PTEN-ASD or would also be observed in PTEN no-ASD. Intriguingly, there were no significant group differences in AKT or ERK levels. Re-evaluating PTEN-related pathway protein levels in a new cohort is crucial to determining whether prior exploratory findings replicate and whether other canonical PTEN pathway proteins show abnormal expression patterns.

Variable phenotypes in *PTEN* mutation cases also imply that mutational status alone is not sufficient to predict outcome. Rather, the complexity of PTEN-influenced mechanisms, such as gene–gene and gene–environment interplay, may require multivariable modeling to predict neurobehavioral outcomes. This possibility is consistent with the growing recognition of the importance of primary and secondary factors in determining phenotypic outcomes in ASD cases, even when rare pathogenic variants are identified [36, 37]. An important advance of the present study is inclusion of PTEN no-ASD cases to evaluate whether combinations of protein levels may predict phenotypic outcomes beyond *PTEN* mutation status. The present study also compares key protein differences observed in human ASD and/or PTEN mutation cases with neural protein expression data from a knock-in murine *PTEN* model (m3m4).

### The present study

The present study evaluated the influence of *PTEN* mutation and ASD diagnostic status on relevant pathway protein levels in peripheral blood. In addition to understanding pathophysiology, our study also begins the process of identifying peripheral markers that may reflect the brain. Peripheral PTEN levels were hypothesized to be lower in all *PTEN* mutation cases, and downstream signaling molecules such as P-S6 and P-AKT were predicted to be upregulated in individuals with germline *PTEN* mutations without ASD. No specific predictions were made for P-S6/S6/P-AKT levels in the ASD with or without *PTEN* mutation groups. The secondary purpose was to investigate cross-level relationships between pathway measures and neurobehavioral domains independent of mutational or diagnostic status. We hypothesized that PTEN and S6 levels would be significantly associated with global cognitive ability, but no specific predictions were made for other proteins or neurobehavioral measures. Finally, the study also explored whether protein levels, as a group, might predict neurobehavioral functioning beyond *PTEN* mutation status.

## Methods

### Participants

Participants in this study were recruited from four large tertiary medical centers (Cleveland Clinic, Boston Children’s Hospital, Stanford University, and University of California, Los Angeles) as part of an IRB-approved, ongoing, multicenter prospective study designed to examine the natural history of autism and germline heterozygous *PTEN* mutations. All potential study participants were screened by a clinical psychologist with expertise in ASD to determine whether they met Diagnostic and Statistical Manual of Mental Disorders—Fifth Edition (DSM-5) diagnostic criteria for ASD. All potential participants also underwent genetic testing to determine the presence/absence of a mutation in *PTEN*. Individuals were included in the study if they met the following criteria: (1) age 3–21 years; (2) confirmed diagnosis of ASD (based on consensus of expert clinician evaluation and Autism Diagnostic Observation Schedule-2) and/or a confirmed heterozygous mutation in *PTEN*; (3) English as primary communicative language; and (4) completion of baseline neuropsychological evaluation. In addition, individuals with ASD and macrocephaly, but without a *PTEN* mutation (Macro-ASD), had to have an orbitofrontal head circumference ≥ 98th percentile for their age to be included in the study. Informed consent for study participation was obtained from adult participants and/or a parent or legal guardian. Assent was obtained from all participants age 7 years and older who were cognitively able to provide the same.

A total of 84 participants met initial study inclusion criteria, and of these, 68 had PTEN pathway protein measurements with at least one neurobehavioral test result. However, 7 cases (2 PTEN-ASD, 3 PTEN no-ASD, and 2 Macro-ASD) had invalid protein measurement results that were more than 6 standard deviations beyond the remaining values and were therefore excluded. The final sample included 61 participants: 25 PTEN-ASD (ASD with macrocephaly, *PTEN* mutation), 16 PTEN-no ASD (no ASD, *PTEN* mutation), and 20 Macro-ASD (ASD with macrocephaly, no *PTEN* mutation).

### PTEN genotyping

Germline genomic DNA was extracted in the Genomic Medicine Institute’s Genomic Medicine Biorepository. PCR-based LightCycler mutation scanning and semi-automated PCR-based Sanger sequencing (ABI3730xl in Genomics Core Facility) of exons 1 through 9 and flanking intronic regions of genomic DNA were performed as per routine in the Eng laboratory since 1997 to reveal germline intragenic mutations in exons 1–9 and in splice sites. All novel variants were checked for presence and frequency in 350 ancestry-matched, sex-matched population controls available in the Eng laboratory, ClinVar-PTEN, 1000G, and gnomAD.

### PTEN pathway protein assays

**Fig. 1:**
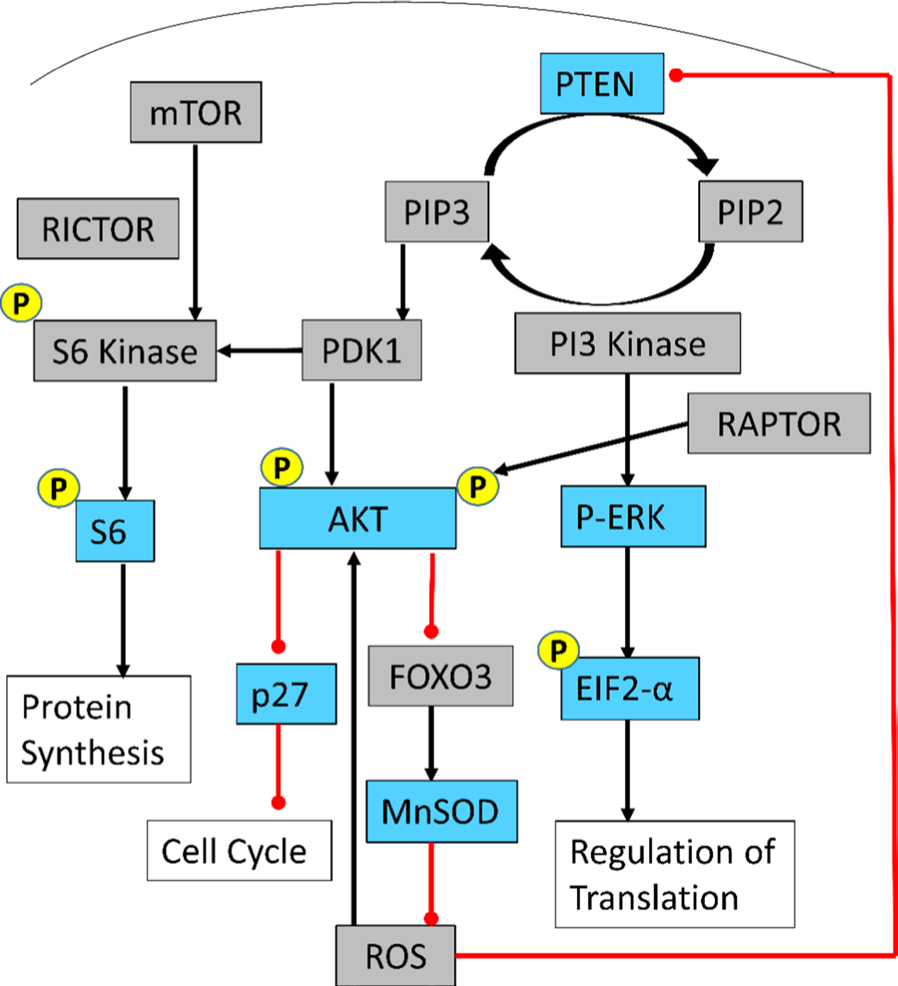
Canonical PTEN pathway depicting PI3K/AKT/mTOR and MAPK/ERK signaling

Western blot analyses of lymphoblastoid cell total protein lysate were performed with the antibodies and techniques routinely used in the Eng laboratory, by investigators blinded to subject genotype and group [38]. Protein lysates were prepared according to Genomic Medicine Biorepository standards (https://www.lerner.ccf.org/gmi/gmb/). All lysates were quantified for protein content using BCA assays, and equalized for protein content, and 40 μg of protein per sample was loaded on a 10% TGX polyacrylamide gel. The separated proteins were transferred to a polyvinylidene difluoride (PVDF) membrane, and the membrane was blocked overnight in 3% normal goat serum at 4 °C. Membranes were then washed with Tris-buffered saline, containing 0.2% Tween-20 TBST, and incubated with experiment-specific primary antibodies diluted in TBST overnight at 4 °C. The following antibodies were used: PTEN (1:1000, clone 6H2.1, #ABM-2025, Cascade Bioscience, Winchester, MA), P-AKT-S473 (1:1000, #4060L, Cell Signaling) and total AKT (1:750, # 4691, Cell Signaling), P-ERK1/2 (1:1000, #4376, Cell Signaling) and total ERK1/2 (1:750, #9102, Cell Signaling), P-S6-S235/236 (1:2000, #2211, Cell Signaling) and total S6 (1:1000, #2217, Cell Signaling), p27 KIP1 (1:1000, #3686, Cell Signaling), EIF2 (1:1000, #9722, Cell Signaling), MnSOD (1:1000, #06-984, Millipore, Burlington, MA), GAPDH (1:10,000, #2118L, Cell Signaling), and Actin (1:10,000, sc-8432, Santa Cruz, Dallas, TX). We removed the primary antibody solution and performed three washes, for 10 min per wash, with TBST. Blots were probed with Goat anti-Rabbit or antimouse secondary antibodies conjugated with horse radish peroxidase (1:10,000 or 1:20,000, Southern Biotech, Birmingham, AL) diluted in TBST, for 2 h at room temperature. The PVDF membranes were washed three times, 10 min each in TBST, and imaged using the iBright FL1000 (Invitrogen, Carlsbad, CA). Using ImageJ (National Institute of Health, Bethesda, Maryland, 1995), we performed densitometry analysis on these images to quantify protein expression. Active PTEN results in decreased phosphorylation of AKT and ERK, upregulation of p27, and downregulation of cyclin D1 protein levels, resulting in decreased proliferation and increased apoptosis (canonical PTEN signaling—Fig. 1). All of these proteins have been shown to be consistent in peripheral protein lysates from 100 population controls [38]. Thus, protein studies should reveal the canonical functional effects of *PTEN* alterations.

Western blots using protein lysates from mouse brains and livers for murine downstream protein expression were performed as previously reported [39, 40]. The PTEN-m3m4 murine model has been reported [29, 39, 40].

### Neurobehavioral measurements

Neurobehavioral assessment included a cognitive battery consisting of developmentally appropriate measures of global, verbal, and non-verbal cognitive ability, language, attention, working memory, and processing speed. Due to anticipated severe symptom and intellectual impairment in at least a subset of cases, cognitive ability, language, working memory, and processing speed measures were prioritized for completion. Parents/guardians completed questionnaires designed to measure adaptive function, motor difficulties, autism symptoms, and behavioral challenges [35]. The specific cognitive and behavioral measures used in this study are outlined in Additional file 1: Table S1. All measures were scored according to published test manuals using age- and/or sex-corrected norms as available. To reduce the number of analyses and focus on domains rather than specific measures, language, attention, and motor measures were combined into separate composite scores for each domain. A frontal sub-cortical composite was also created based on our prior data. This composite averaged the attention, working memory, processing speed, and motor measures into a single broad composite score.

**Table 1:** Sample characteristics

	(1) Macro-ASD	(2) PTEN no-ASD	(3) PTEN-ASD	*F*/*Χ*^2^ (*p*)	1 vs. 2	1 vs. 3	2 vs. 3
	M (SD)	M (SD)	M (SD)		*d*	*d*	*d*
N	20	16	25				
Age	13.9 (4.7)	9.3 (4.3)	9.5 (5.2)	5.71 (.005)	*1.02*	*.88*	− .04
Male (n, %)	17 (85%)	10 (63%)	20 (80%)	2.75 (.252)	.54	.12	− .39
Race/ethnicity				15.72 (.108)	− .63	− .39	.24
White non-Hispanic	9 (45%)	12 (75%)	16 (64%)				
Hispanic	1 (5%)	0 (0%)	5 (20%)				
Black	1 (5%)	0 (0%)	1 (4%)				
Asian	4 (20%)	1 (6%)	0 (0%)				
Other/unreported	5 (25%)	3 (19%)	3 (12%)				
Head circumference (cm)	59.2 (3.0)	58.8 (1.9)	59.9 (2.8)	0.82 (.445)	.16	− .14	− .32
Height (cm)	147.6 (18.8)	150.0 (18.1)	144.3 (17.8)	0.54 (.586)	− .13	.18	.32
Weight (kg)	50.1 (15.5)	40.7 (14.7)	48.3 (14.5)	1.90 (.160)	.62	.12	− .52
Systolic BP	117.4 (14.9)	110.1 (14.0)	108.8 (13.5)	1.93 (.157)	.50	.60	.09
Diastolic BP	71.6 (14.9)	64.1 (14.4)	65.7 (14.5)	1.32 (.275)	.51	.40	− .11
Heart rate (bpm)	91.7 (14.4)	94.7 (13.8)	91.6 (13.5)	0.16 (.855)	− .21	.01	.23
Respiration (cycle/min)	18.4 (3.8)	19.5 (4.1)	20.4 (3.6)	2.68 (.081)	− .28	− .54	− .23
Temperature (°F)	98.1 (1.0)	98.0 (1.3)	97.8 (1.1)	0.96 (.388)	.09	.29	.17

Italicized Cohen’s d value indicates a significant group difference (*p* < .05). For qualitative variables, row proportion differences were used to calculate the effect size. For race/ethnicity effect size was calculated as white non-Hispanic vs. other race/ethnicities

Incomplete neurobehavioral data were largely due to a subset of patients who were unable to tolerate testing or due to time constraints during research visits. For those patients who could not complete a particular measure due to low functional capacity, the lowest possible score was assigned [33, 35]. Assessments that were incomplete due to time constraints were considered missing at random. Missing data (10%) were minimal for intelligence quotient (IQ), frontal sub-cortical composite, and language composite measures (< 4%) but higher for attention (36%) and processing speed (28%). Missing data were more likely to be present in younger children (*r* = 0.28–0.30), and attention data were more likely to be missing in *PTEN* patients (*r* = 0.31), potentially reflecting greater attention impairment leading to the test not being attempted. There were no relationships between missing data and sex, race, ASD diagnosis, or general cognitive ability (IQ). To address missing data, five imputed datasets were computed using the SPSS missing values analysis option with a fully conditional specification using Markov Chain Monte Carlo estimation. All variables were included in the multiple imputation process to satisfy the missing at random assumption, to reduce bias, and to increase the precision of imputed scores [41–43]. Results were highly consistent between the original and imputed datasets. For this reason, neurobehavioral results are presented using the first imputation dataset.

### Statistical analyses

Frequency distributions and bivariate plots were evaluated, but no univariate or bivariate outliers or high leverage cases were detected. To characterize the sample, univariate analyses of variance and Chi-square analyses compared patient groups across demographic (age, sex, race/ethnicity), clinical (head circumference z-score, height, weight, heart rate, respiration, body temperature, and systolic and diastolic blood pressure), and neurobehavioral measurements. For clinical and neurobehavioral measurements, age and sex were included as covariates, as age and sex were not equivalent across groups and were correlated with clinical and neurobehavioral variables. Protein assay scores were compared across groups using analysis of variance, and the independent effects of *PTEN* mutation status and ASD diagnosis were evaluated in linear regression models. *PTEN* mutation (yes/no) and ASD (yes/no) were predictors with protein scores as dependent variables in separate analyses. For these analyses, raw protein scores were the primary measures, but we also explored ratio scores for P-AKT/AKT, P-ERK/ERK, and P-S6/S6.

To examine cross-level relationships between protein assays and neurobehavioral measures, we first examined the bivariate correlation between PTEN levels and global cognitive ability as we predicted a positive association based on our prior findings. Second, all correlations between protein assays and neurobehavioral measures were computed. To examine whether this matrix reflected random associations or whether the number of statistically significant correlations exceeded the expected number based on chance a sign test was computed. A significant value of this test would indicate that at least some of the significant observed correlations were not due to change and likely represent meaningful associations. Significant correlations were interpreted with reference to expected canonical influences of *PTEN* mutations. Observed relationships were then tested in structural (path) models to better understand these relationships. Specifically, mediational models were computed if any patterns of correlation suggest potential mediation by downstream (S6, EIF2A, p27) molecules of relationships between canonical upstream pathway proteins (PTEN, AKT, ERK) and neurobehavioral measures.

To evaluate whether PTEN-related pathway proteins improve prediction of neurobehavioral measures (as a group) over and above *PTEN* mutation status, a series of hierarchical linear regressions were computed with *PTEN* mutation (yes/no) entered in Step 1 and forward entry (*p* < 0.05, removal *p* > 0.10) of protein assay values in Step 2. A significant increment in *R*^2^ would indicate that protein measure improves prediction over and above mutation status. In this case, stepwise regression provides useful information about which protein measures enter the equation and provide a significant prediction of neurobehavioral outcomes. However, stepwise regression tends to overfit a dataset and will often produce unrealistically large predictive values. For this reason, we also computed a support vector machine regression model with *PTEN* mutation (yes/no) and all protein assay values entered as predictors of neurobehavioral measures. A fivefold cross-validation was used to evaluate predictive validity of each model. While less optimal than having a holdout test sample, which is not possible in this modest rare disease cohort, this approach should produce more realistic estimates of validity generalization.

Sensitivity power analysis indicated that a large effect size (*f* ≥ 0.41, d = 0.82) was required to have at least good power (1-*β* ≥ 0.80) to detect statistically significant group differences (*p* < 0.05, two-tailed). For correlational analyses, given a total sample size of 61, a correlation (*r* ≥|.31|) was required to have adequate power (1-*β* ≥ 0.70) to detect a significant association. Power to detect structural model parameters, including indirect (mediation) effects, is approximately equivalent to the power for bivariate correlations [44]. Following the a priori prediction that PTEN levels would be lower in mutation cases, a one-tailed *p* < 0.05 was used to evaluate this effect. For all other statistical tests, two-tailed *p* < 0.05 was used. Significant *R*^2^ (*p* < 0.05) from fivefold cross-validation was used to identify models with significant predictive validity for neurobehavioral measures. No correction was made for multiple comparisons given the small sample size and preliminary nature of these analyses. Statistical analyses were computed using SPSS version 27 [45]. Mediational models were computed using MPlus version 7.2 [46], following the analytic criteria described by Muthén, Muthén, and Asparouhov [44].

## Results

### Sample characteristics

Macro-ASD patients were significantly older than PTEN-ASD and PTEN no-ASD patients (Table 1). There were no significant differences in sex distributions, although PTEN no-ASD tended to have lower proportions of males than the other ASD groups, consistent with the known sex ratio in ASD [47]. There were no significant differences in race/ethnicity or any other clinical variable (all *p* > 0.08). All PTEN patients had significant macrocephaly. A summary of the germline PTEN variants observed in study groups, including fitness [48] and abundance [49] scores for missense mutations, is provided in Additional file 1: Table S2.

**Table 2:** PTEN pathway protein levels across groups and by ASD and PTEN status

	(1) Macro-ASD	(2) PTEN no-ASD	(3) PTEN-ASD	*F* (*p*)	*R*^2^	ASD	PTEN
	*M* (SD) *n* = 20	*M* (SD) *n* = 16	M (SD) *n* = 25			*β* (*p*)	*β* (*p*)
PTEN	140.5 (41.7)	121.1 (50.8)	116.4 (47.4)	1.59 (.213)	.05	− .04 (.754)	− **.24 (.045)**
P- AKT	115.3 (42.0)	126.5 (36.2)	122.1 (38.5)	0.38 (.686)	.01	− .05 (.722)	.08 (.568)
Total AKT	133.4 (75.3)	139.1 (64.3)	126.3 (64.9)	0.18 (.838)	.01	− .08 (.561)	− .05 (.730)
P- ERK	110.0 (57.0)	130.0 (67.8)	105.3 (54.2)	0.90 (.411)	.03	− .19 (.196)	− .04 (.790)
Total ERK	195.4 (79.5)	194.5 (70.1)	183.9 (72.5)	0.17 (.847)	.01	− .06 (.657)	− .08 (.606)
IGFBP2	166.1 (41.3)	180.4 (41.7)	156.5 (54.4)	1.25 (.295)	.04	.22 (.120)	− .69 (.498)
MnSOD	175.5 (48.9)	202.2 (41.0)	160.5 (46.9)	**4.00 (.024)**	.12	− **.38 (.006)**	− .15 (.284)
P- S6	94.6 (58.6)	67.1 (33.8)	115.5 (59.3)	**3.54 (.035)**	.11	**.36 (.010)**	.17 (.226)
S6	160.4 (47.6)	145.2 (52.0)	131.7 (43.4)	2.07 (.136)	.07	− .13 (.373)	− **.28 (.047)**
EIF2A	17.8 (5.1)	19.9 (4.1)	19.9 (4.5)	1.54 (.222)	.05	− .01 (.962)	.22 (.120)
p27	13.7 (5.7)	16.4 (6.6)	12.8 (7.4)	1.43 (.248)	.05	− *.24 (.100)*	− .07 (.649)
P-AKT/AKT	1.22 (1.11)	1.17 (0.84)	1.22 (0.64)	0.02 (.978)	< .01	.05 (.848)	< .01 (.999)
P-ERK/ERK	0.87 (0.95)	0.91 (0.90)	0.80 (0.69)	0.10 (.903)	< .01	− .12 (.666)	− .08 (.770)
P-S6/S6	0.64 (0.44)	0.52 (0.27)	0.88 (0.42)	4.40 (.017)	.13	**.36 (.007)**	**.24 (.045)**

All protein levels are 10-e + 3. Bold designates *p* < .05, and italics designates *p* ≤ .10. Per a priori hypothesis, a one-tailed *p *value was used for evaluating the effect of *PTEN* mutations status on PTEN protein levels

### Protein levels

**Fig. 2:**
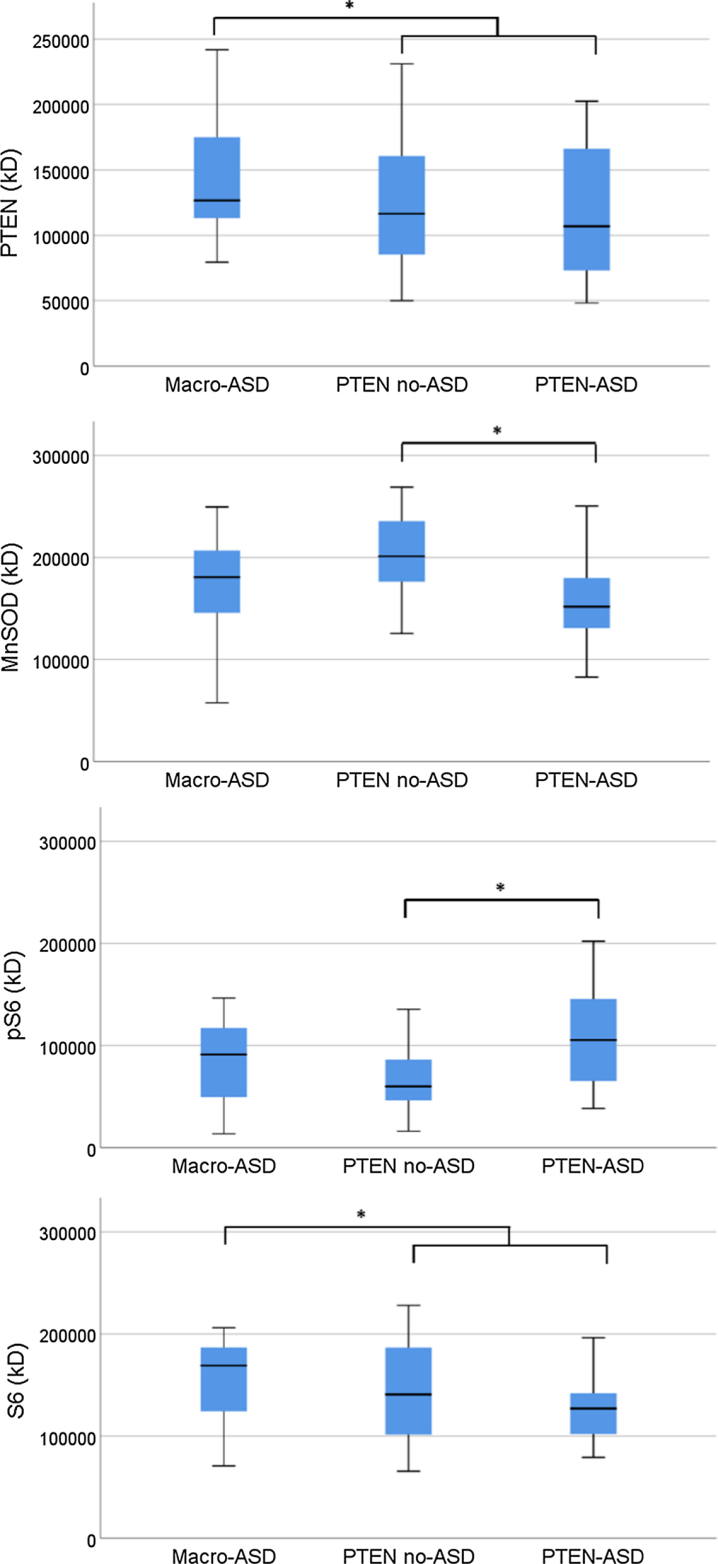
Boxplots for PTEN, MnSOD, P-S6, and S6. *p < .05 for group comparisons. kD = kilo Dalton

Significant group differences were observed for MnSOD, P-S6, and the ratio of P-S6/S6 (Table 2). The presence of genotype-independent ASD was associated with reductions in MnSOD and increases in P-S6 and P-S6/S6. *PTEN* mutation status was associated with reduced PTEN levels (as hypothesized), reduced S6 (Fig. 2), and increased P-S6/S6 ratio. Trends toward reduced p27 with the presence of genotype-independent ASD with *PTEN* mutation status were also observed. The magnitude of *PTEN* and ASD effects tended to be medium-sized (*β* =|.24–0.38|), even when simultaneously controlling for the other predictor.

### Murine model PTEN pathway findings

**Fig. 3:**
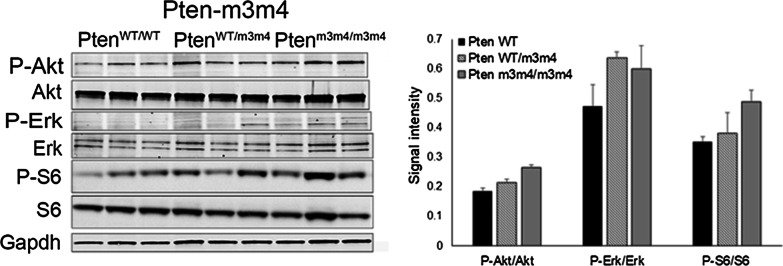
(Left) Western blots on murine brain tissue at 6 weeks of age showing levels of PI3K pathway mediators in a model of *PTEN* mutation. (Right) Quantification of signal intensity for each mediator normalized to Gapdh (*n* = 3 each)

*PTEN-*m3m4 murine models showed PI3K/AKT pathway activation (homozygous > heterozygous > wildtype), including increased phosphorylation of Akt and S6, in brain (Fig. 3) and liver (data not shown). However, variability was substantial, particularly in heterozygous mice. Together, these findings suggest canonical PTEN pathway activation, but also substantial variability within and across models, particularly in the heterozygous mice which most closely mirror heterozygosity in the human condition.

### Neurobehavioral differences

Consistent with our prior analyses [35], large group differences were observed across IQ (full-scale, verbal, and non-verbal), frontal sub-cortical, motor, and adaptive behavior measures (Additional file 1: Table S3). PTEN-ASD patients were impaired across all domains with severe impairments in IQ, frontal sub-cortical (attention, working memory, processing speed, motor), and adaptive functions. PTEN no-ASD had generally intact cognitive ability, but showed small average reductions in frontal sub-cortical measures (with the exception of working memory). PTEN no-ASD also had slightly elevated internalizing problems and autism traits relative to population expectation. These data support wide neurobehavioral variability in *PTEN* mutation cases, indicating that mutation status alone does not account for phenotypic presentation.

### Cross-level relationships

Higher PTEN levels were significantly associated with lower general cognitive ability (*r* = -0.26, *p* = 0.024). Overall, the number of statistically significant correlations (*p* < 0.05 or *p* < 0.10) between the molecular and neurobehavioral measures was greater than would be expected by chance (*p* = 0.010 for *p* < 0.05 threshold and *p* = 0.003 for *p* < 0.10 threshold) (Additional file 1: Table S4). The major patterns observed were negative correlations between PTEN protein levels and IQ, language, frontal sub-cortical, working memory, and adaptive functions; positive correlations between total AKT and EIF2A levels and a highly overlapping set of neurobehavioral functions to that observed for PTEN; and positive correlations between p27 levels and frontal sub-cortical, attention, processing speed, and motor functions. For protein ratios, only 3 significant correlations were observed—a negative relationship between P-AKT/AKT ratio and autism traits and positive relationships between P-S6/S6 ratio and externalizing problems and repetitive behavior (Additional file 1: Table S5).

**Fig. 4:**
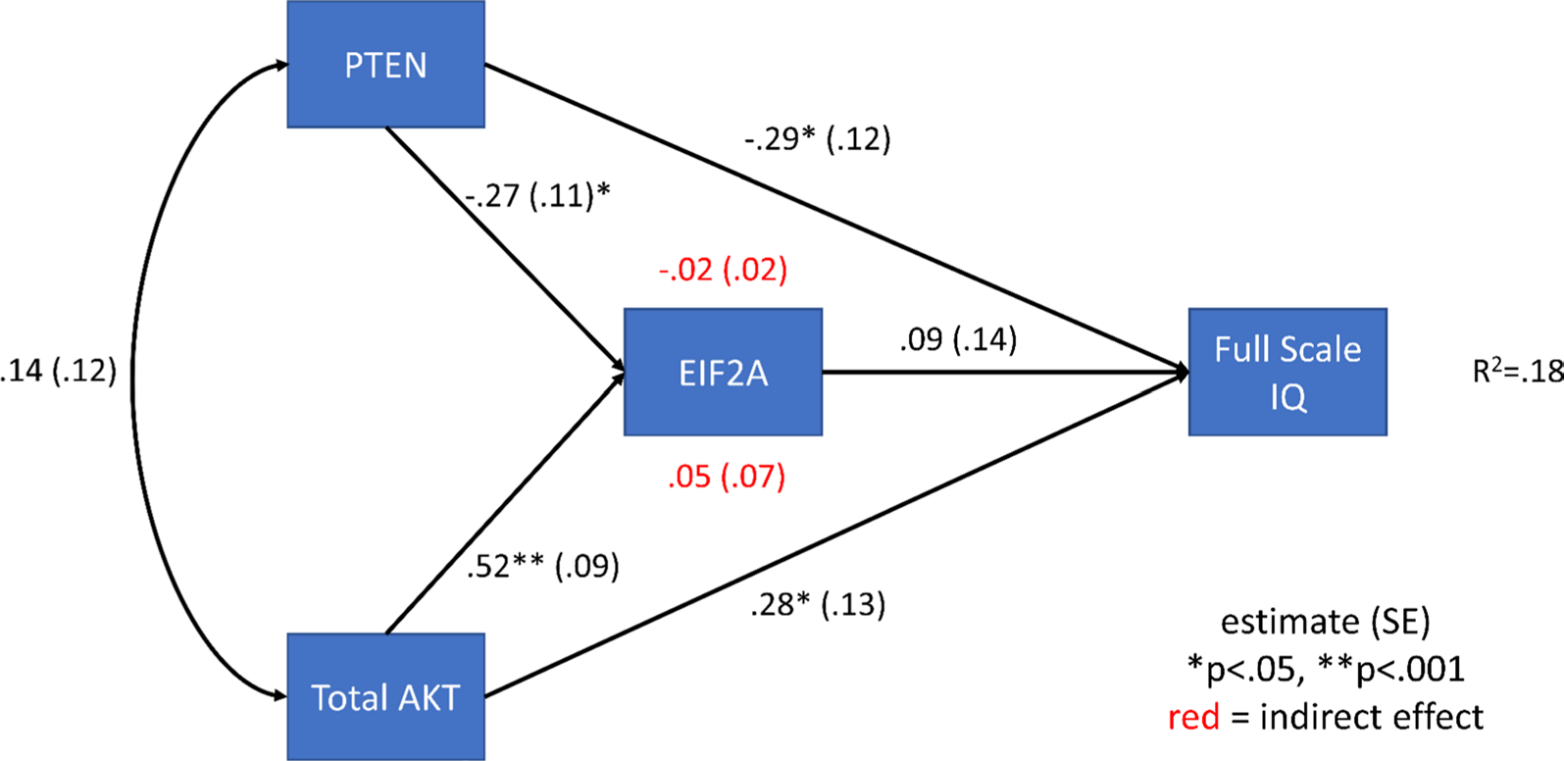
Effects of PTEN and total AKT protein levels on full-scale IQ directly and indirectly via EIF2A

To further understand how PTEN, AKT, and EIF2A levels may interact with global IQ differences, a structural mediational model was computed with direct effects of PTEN and AKT on full-scale IQ and indirect effects through EIF2A being estimated. This model was chosen because of the well-known multiple functions of PTEN beyond AKT pathway regulation and the known downstream impact on EIF2A influencing its role in transcription. PTEN and AKT showed significant direct effects on full-scale IQ, but the indirect effects through EIF2A were small and not significant (Fig. 4). p27 significantly influenced frontal sub-cortical and motor function and marginally significantly influenced attention, when these relationships were simultaneously estimated (Additional file 1: Fig. S1).

*PTEN* mutation status did not account for significant variance in any of the neurobehavioral measures. However, both in the regression and support vector models, protein measures significantly predicted IQ scores, language, frontal sub-cortical composite, and externalizing scores. Using fivefold cross-validation, the magnitude of predictive validity was medium-to-large (*r* = 0.26–0.47) across these domains (Table 3).

**Table 3:** Hierarchical regression and support vector machine models with *PTEN* mutation status and protein assays predicting neurobehavioral measures

	Hierarchical regression				Support vector machine
	Predictors retained beyond PTEN mutation status	PTEN mutation	Protein levels		PTEN mutation and protein levels
		*R*^2^	Δ*R*^2^	Δ*F* (*p*)	Fivefold cross-validation *R*/*R*^2^
Full-scale IQ	PTEN, total AKT	< .01	.17	5.90 (.005)	.33/.11*
Verbal IQ	PTEN, total AKT, MnSOD	< .01	.16	4.97 (.004)	.35/.12*
Non-verbal IQ	EIF2A	< .01	.08	4.73 (.034)	.28/.08*
Language composite	EIF2A	< .01	.09	5.57 (.022)	.27/.08*
Frontal-subcortical composite	PTEN, p27	< .01	.16	5.56 (.006)	.26/.07*
Attention composite	p27	.02	.07	4.72 (.034)	.14/.02
Working memory	–	.02	–	–	.37/.14*
Processing speed	–	.01	–	–	.20/.04
Motor skills composite	p27	.01	.10	6.48 (.014)	.01/.01
Internalizing problems	–	< .01	–	–	.24/.06
Externalizing problems	P-AKT, P-S6, S6	.02	.20	4.67 (.006)	.47/.22**
Autism traits	–	.03	–	–	.17/.03
Repetitive behavior	Total P-ERK, P-S6	< .01	.20	4.66 (.006)	.22/.05
Adaptive behavior	–	.01	–	–	.10/.01

Hierarchical regression models included *PTEN* mutation status in step 1 and all protein levels in step 2, with specific predictor entry set at *p* < .05 and removal at *p* > .10. Support vector models included *PTEN* mutation status and all protein assays in separately predicting each neurobehavioral variable. Support vector model fivefold cross-validation *R* significance based on full sample. *p < .05, **p < .001

## Discussion

PTEN levels were modestly but reliably reduced in PTEN-ASD and PTEN no-ASD groups, replicating and extending our prior findings from a PTEN-ASD only cohort [33]. This modest, but variable, reduction likely reflects the inclusion of many missense mutation cases where PTEN levels may not be substantially reduced. This may suggest that early developmental timing of alterations in PTEN protein levels is a key factor in determining neurobehavioral outcomes. It is also possible that the negative correlations observed between PTEN protein levels and cognitive ability and language reflect a dominant negative process operating in some cases where one mutant *PTEN* allele’s product negatively impacts the remaining wildtype PTEN protein to disrupt neurodevelopment. Specifically, in missense mutations where PTEN protein levels are not reduced, mutant PTEN may still bind substrate but not be able to act on it. This could trap substrate and prevent it from being catalyzed by wildtype PTEN. Additionally, PTEN functionally exists as dimers [50]. In heterozygotes, mutant PTEN dimerizes with wildtype PTEN protein and prevents the latter from working on substrate. If a dominant negative process is operating, it may suggest that early interventions that focus on degrading mutant PTEN could be a useful strategy in some cases. Recent work has demonstrated substantial variability in impact on PTEN protein stability and function across different missense mutations [48, 49, 51]. This suggests that the above-described pattern of modest reduction in PTEN levels may mask substantial variability, potentially explaining the observed heterogeneity of outcomes across PTEN mutation cases. Future work in large, longitudinal cohorts is needed to better understand the precise developmental timing and mechanisms—including variability in stability and function across missense mutations—leading to ASD and related neurobehavioral phenotypes in PTEN patients.

The direct relationships between PTEN and total AKT levels with cognitive ability indicate that the canonical PI3K/AKT/mTOR pathway may play an important role in neurobehavioral variation within *PTEN* mutation-positive patients [52]. This possibility is consistent with mouse model findings [28, 29], although both murine and human results suggest high variability in the effects on PI3K/AKT/mTOR activation [29, 33, 35]. In spite of this variability, the statistically reliable identification of canonical PI3K/AKT/mTOR activation and its relationship with cognitive ability suggests that modulation of this pathway may be an effective intervention for neurobehavioral impairments. In fact, the elevation of P-S6 would implicate mTORC1, hence, the potential efficacy of mTOR inhibition. The ongoing multisite randomized controlled trial of RAD001 (Everolimus) for PTEN-associated neurodevelopmental disorders will be an important test of this hypothesis (NCT02991807). However, it is also important to note that, in this study, the effects of PTEN and AKT were only minimally mediated by EIF2A and not mediated by other downstream molecules. Thus, it will be important to further explore the exact molecular and neural mechanisms by which PTEN and AKT protein levels influence cognitive ability and language and the developmental timing of these mechanisms.

The variability in PTEN downstream molecules both in human peripheral blood and in the PTEN-murine heterozygote model brain, in spite of a highly conserved genetic background and consistent environment, suggests that other non-PTEN influences may interact with the *PTEN* mutation to ultimately affect phenotype. We have recently shown that CNV load may interact with germline *PTEN* mutations to favor ASD over cancer phenotypes [53]. Similarly, the metabolic milieu may also crosstalk with the *PTEN* mutation, in a phenotype-specific manner [54]. We already appreciate the complexity of ASD even with the contribution of the genetic underpinnings. Modifier effects, as they crosstalk with Mendelian genes, will become germane in human ASD predisposed by germline mutations in Mendelian genes [36].

Decreases in MnSOD and elevations in P-S6 in ASD cases, irrespective of genotype, are consistent with prior molecular findings in idiopathic ASD [55–59]. Specifically, MnSOD is a redox catalyst, located in the mitochondrial matrix, and plays a role in many biological processes to reduce O2 and producing an influx of O2˙radicals. Decreased levels of MnSOD have been associated with increased oxidative stress in idiopathic ASD [59]. Prior research has suggested that subsets of idiopathic ASD cases show oxidative stress [55, 56] and mitochondrial dysfunction [57, 58] and metabolomic algorithms have shown promise in differentiating ASD and healthy control cases [60]. Furthermore, increased oxidative stress has been associated with muscle function [61] and, in the current investigation, both ASD groups had substantially decreased motor functioning, a well-replicated finding in idiopathic ASD [62–64]. The potential to impact at least the motor portion of the *PTEN* mutation neurobehavioral phenotype via improving the functioning of oxidative stress pathways should be explored in future research and could represent an augmentative strategy for future clinical trials.

The increases in P-S6 observed in ASD groups within this study are congruent with the notion of increased protein translation as a converging signaling mechanism within at least some ASD cases [36, 65, 66]. Altered protein translation also provides a molecular mechanism for excessive early life head growth [67, 68], often resulting in macrocephaly [69, 70], within a subset of ASD. Alternatively, or possibly simultaneously, increased P-S6 may contribute to dysfunctional energy metabolism associated with abnormal mTOR [52, 71] or insulin signaling pathway activation [72]. Based on these findings, it is plausible that MnSOD and P-S6 could serve as early peripheral markers of ASD within *PTEN* mutation cases and potentially in children with excessive early life head growth and/or macrocephaly who are at higher risk for ASD, while preliminary, the observation of upregulation of phosphorylated AKT and S6 from both peripheral blood protein lysate and PTEN model murine brains and livers, lends hope that at least some PTEN signaling molecules in the periphery do mirror those in the brain.

As a group, canonical PTEN pathway proteins significantly predicted several neurobehavioral domains, including cognitive ability, language, frontal sub-cortical function, and externalizing behavior. Larger samples and future waves of longitudinal data collection will be useful for replicating these effects and determining which proteins are playing important roles. Regardless, these findings imply that canonical PTEN pathways are an important part of understanding and predicting the variability in neurobehavioral outcomes in *PTEN* mutation patients. Beyond replication and clarification of these findings, future studies should search for other non-canonical mechanisms, as there is substantial variance remaining to be explained. If replicated, and additional factors are identified with useful predictive validity, an aggregate index of peripheral molecular markers could be a useful advance for early prediction of neurobehavioral outcome and would allow for better planning and tailoring of early intervention strategies. This index might also have value for longitudinal tracking, including evaluating and understanding target engagement and efficacy within clinical trials.

### Limitations

While reasonable for a rare genetic syndrome, sample sizes in the present study were small and precluded analysis of cross-level relationships within each patient group. This step is critical before final conclusions are made since relationships observed might be driven by the specific pathophysiology observed in one group and not the other. A key task of future longitudinal waves will be to retain existing patients while adding new patients to the cohort. These waves would also be wise to add protein and other molecular assays for non-canonical PTEN effects. This will increase the power and explanatory value of future molecular and cross-level analyses and could improve the validity of an aggregate molecular index in predicting neurobehavioral outcomes.

An additional limitation was the lack of additional time points to provide both replication and evaluation of stability versus change in protein levels, neurobehavioral functions, and their inter-relationship. Furthermore, lack of neural systems measures leaves an important gap for evaluating how protein measures might influence neural structure and function to produce neurobehavioral effects. Adding intermediate neural system measures that can be readily acquired in all PTEN patients, such as resting electroencephalogram or event-related potential paradigms, would provide key information between molecular assays and neurobehavioral outcomes and could clarify the full picture of how *PTEN* mutations result in widely disparate phenotypic outcomes.

Samples sizes were also insufficient to evaluate the functional impact of missense mutations in PTEN patients [51]. Future work in larger samples, computing fitness [48] and abundance [49] scores and identifying candidate dominant negative mutations, will be crucial to understanding whether different mutations influence peripheral protein levels and/or neurobehavioral outcomes.

### Conclusions

The present study identified several protein-level group differences and relationships between PTEN pathway proteins and neurobehavioral functions that clarify how germline heterozygous *PTEN* mutations may impact neurobehavioral function. If replicated, these protein changes could not only improve understanding of individual differences in neurodevelopment within *PTEN* mutation cases but could also serve as peripheral markers of key neurobehavioral outcomes.

## Supplementary information

**Additional file 1.**

## Data Availability

The data generated and/or analyzed for the current study are not publicly available due to an embargo period, but, when this period has terminated, data will be made available by the RDCRN upon reasonable request.

## Abbreviations

ASD: Autism spectrum disorder
DSM-5: Diagnostic and Statistical Manual of Mental Disorders—Fifth Edition
IQ: Intelligence quotient/global cognitive ability
Macro-ASD: Patients with macrocephalic autism spectrum disorder without *PTEN* mutations
PTEN-ASD: Patients with *PTEN* mutations and autism spectrum disorder
PTEN no-ASD: Patients with *PTEN* mutations without autism spectrum disorder
PVDF: Polyvinylidene difluoride
