# Supplementary material for: Cross-level analysis of molecular and neurobehavioral function in a prospective series of patients with germline heterozygous PTEN mutations with and without autism
Source: Mol Autism. 2021 Jan 28;12:5. doi: 10.1186/s13229-020-00406-6 (PMC7841880; doi:10.1186/s13229-020-00406-6)
Supplement: Supplementary file 1 — Additional file 1. [file 13229_2020_406_MOESM1_ESM.docx]

**Supplemental Table S1.** Specific cognitive and behavioral measures and the construct evaluated.

| Construct | Measure(s) | Type | Scale |
| --- | --- | --- | --- |
|  |  |  |  |
| Global Cognitive Ability | Full Scale IQ* | Clinician-administered | SS |
| Verbal Cognitive Ability | Verbal IQ* | Clinician-administered | SS |
| Non-Verbal Cognitive Ability | Non-verbal IQ* | Clinician-administered | SS |
| Language composite | Peabody Picture Vocabulary Test – Fourth Edition  Expressive Vocabulary Test – Second Edition  Verbal IQ  Vineland Adaptive Behavior Scale - Second Edition  Communication domain | Clinician-administered  Clinician-administered | z |
| Frontal-subcortical composite | Attention composite (see below)  Working memory (see below)  Processing speed (see below)  Motor skills composite (see below) | Computer-administered  Clinician-administered  Clinician-administered  Parent-report | z |
| Attention composite | Continuous Performance Test – Third Edition  Omissions, Commissions, Hit Reaction Time,  and Detectability | Computer-administered | z |
| Working memory | Stanford Binet – Working Memory Index | Clinician-administered | z |
| Processing speed | Wechsler Intelligence Scales – Processing Speed Index | Clinician-administered | z |
| Motor skills composite | Developmental Coordination Disorder Questionnaire  Vineland Adaptive Behavior Scale - Second Edition  Motor Skills domain  Beery Visual-Motor Integration test | Parent-report  Parent-report  Clinician-administered | z |
| Internalizing problems | Child Behavior Checklist | Parent-report | T-score |
| Externalizing problems | Child Behavior Checklist | Parent-report | T-score |
| Autism traits | Social Responsiveness Scale-Second Edition total | Parent-report | T-score |
| Repetitive Behavior | Repetitive Behavior Scale – Revised total | Parent-report | Raw score |
| Adaptive Behavior | Vineland Adaptive Behavior Scale – Second Edition  Adaptive Behavior Composite | Parent-report | SS |

**Note.** *IQ scores were derived from the Mullen Scales of Early Learning or Stanford Binet Intelligence Scales-Fifth Edition. Standard scores from the Peabody Picture Vocabulary Test – Fourth Edition and the Expressive Vocabulary Test – Second Edition were average and transformed to z-score metric for ease of interpretation relative to other cognitive composites. Continuous performance test measures included detectability, mean hit reaction time, omissions, and commissions. The Developmental Coordination Disorder Questionnaire raw score was converted to a z-score using available normative data and was averaged with the Vineland Adaptive Behavior Scale – Second Edition Motor scale standard score after this score was also converted to a z-score metric. The Beery Visual-Motor Integration test standard score was included in the motor index because preliminary analysis indicated approximately equal correlations (r~.50) between all three motor composite measures.

**Supplemental Table S2.** *PTEN* mutation and phenotype information for each study group.

| **ID** | **Group** | **Mutation Code** | **Call** | **Type** | **Benign SNP** | **Fitness Score** | **Abundance Score** |
| --- | --- | --- | --- | --- | --- | --- | --- |
| 01-010 | Macro-ASD | Mutation Negative |  |  |  |  |  |
| 01-028 | Macro-ASD | Mutation Negative |  |  |  |  |  |
| 02-004 | Macro-ASD | Mutation Negative |  |  |  |  |  |
| 02-009 | Macro-ASD | Mutation Negative |  |  | c.1026+32T>G |  |  |
| 02-012 | Macro-ASD | Mutation Negative |  |  |  |  |  |
| 03-001 | Macro-ASD | Mutation Negative |  |  | c.1026+32T>G |  |  |
| 03-002 | Macro-ASD | Mutation Negative |  |  | c.1026+32T>G |  |  |
| 03-003 | Macro-ASD | Mutation Negative |  |  | c.1026+32T>G |  |  |
| 03-004 | Macro-ASD | Mutation Negative |  |  | c.1026+32T>G |  |  |
| 03-006 | Macro-ASD | Mutation Negative |  |  |  |  |  |
| 03-008 | Macro-ASD | Mutation Negative |  |  | c.1026+32T>G |  |  |
| 03-009 | Macro-ASD | Mutation Negative |  |  | c.1026+32T>G |  |  |
| 03-010 | Macro-ASD | Mutation Negative |  |  | c.-903G>A |  |  |
| 03-012 | Macro-ASD | Mutation Negative |  |  |  |  |  |
| 03-013 | Macro-ASD | Mutation Negative |  |  |  |  |  |
| 03-015 | Macro-ASD | Mutation Negative |  |  |  |  |  |
| 03-017 | Macro-ASD | Mutation Negative |  |  | c.1026+32T>G |  |  |
| 04-006 | Macro-ASD | Mutation Negative |  |  | c.1026+32T>G |  |  |
| 04-008 | Macro-ASD | Mutation Negative |  |  |  |  |  |
| 04-009 | Macro-ASD | Mutation Negative |  |  | c.1026+32T>G |  |  |
| 01-004 | PTEN no-ASD | c.3G>T | p.Met1Ile | Missense |  | -0.322 | 1.002 |
| 01-009 | PTEN no-ASD | c.388C>T | p.Arg130* | Truncation | c.1026+32T>G |  |  |
| 01-011 | PTEN no-ASD | c.511C>T | p.Gln171* | Truncation |  |  |  |
| 01-012 | PTEN no-ASD | c.97_99delATT | p.Ile33del | Truncation | c.1026+32T>G |  |  |
| 01-015 | PTEN no-ASD | c.740dupA | p.Pro248Thrfs*5 | Truncation | c.1026+32T>G |  |  |
| 01-016 | PTEN no-ASD | c.388C>T | p.Arg130Gly | Missense |  | -2.138 | 1.085 |
| 01-022 | PTEN no-ASD | c.886delT | p.Cys296Varfs*11 | Truncation | c.1026+32T>G |  |  |
| 01-027 | PTEN no-ASD | c.204C>A | p.Tyr68* | Truncation |  |  |  |
| 01-034 | PTEN no-ASD | c.210-2A>G | exon 4 skipping; p.Ala72Thrfs*5 | Splice/truncation |  |  |  |
| 01-036 | PTEN no-ASD | c.955_958delACTT | p.Thr319Terfs | Truncation | c.1026+32T>G |  |  |
| 01-043 | PTEN no-ASD | c.1003C>T | p.Arg335* | Truncation |  |  |  |
| 01-045 | PTEN no-ASD | c.703delG | p.Glu235Lysfs*21 | Truncation | c.1026+32T>G |  |  |
| 01-046 | PTEN no-ASD | c.947delT | p.Leu316Glnfs | Truncation |  |  |  |
| 02-002 | PTEN no-ASD | c.737C>T | p.Pro246Leu | Missense |  | -1.622 | 0.267 |
| 02-006 | PTEN no-ASD | c.494G>T | p.Gly165Val | Missense |  | -2.856 | 0.522 |
| 03-014 | PTEN no-ASD | c.632dupG | p.Cys211Trpfs | Truncation | c.1026+32T>G |  |  |
| 01-001 | PTEN-ASD | c.556_557delCT | p.Leu186fs*3 | Truncation | c.1026+32T>G |  |  |
| 01-002 | PTEN-ASD | c.3G>T | p.Met1Ile | Missense | c.1026+32T>G | -0.322 | 1.002 |
| 01-003 | PTEN-ASD | c.3G>T | p.Met1Ile | Missense |  | -0.322 | 1.002 |
| 01-006 | PTEN-ASD | c.1003C>T | p.Arg335* | Truncation |  |  |  |
| 01-008 | PTEN-ASD | c.494G>T | p.Gly165Val | Missense |  | -2.856 | 0.522 |
| 01-014 | PTEN-ASD | c.697C>T | p.Arg233* | Truncation |  |  |  |
| 01-018 | PTEN-ASD | c.287C>A | p.Pro96Gln | Missense | c.1026+32T>G | -1.784 | 0.390 |
| 01-021 | PTEN-ASD | c.1061C>A | p.Pro354Gln | Missense |  | 0.091 | 1.196 |
| 01-024 | PTEN-ASD | c.277C>T | p.His93Tyr | Missense | c.1026+32T>G | -2.460 | 0.872 |
| 01-029 | PTEN-ASD | c.80-3C>G, c.634_4A>T | Intronic, p.Gly165Ilefs*9 | Splice/truncation | c.-903G>A |  |  |
| 01-031 | PTEN-ASD | c.517C>T | p.Arg173Cys | Missense |  | -1.261 | 0.552 |
| 01-032 | PTEN-ASD | c.385G>A | p.Gly129Arg | Missense | c.1026+32T>G | -3.490 | 0.489 |
| 01-033 | PTEN-ASD | c.103A>G | p.Met35Val | Missense |  | -1.269 | 1.061 |
| 01-035 | PTEN-ASD | c.3G>T | p.Met1Ile | Missense | c.1026+32T>G | -0.322 | 1.002 |
| 01-038 | PTEN-ASD | c.944_945delAT | p.Try315Serfs | Truncation | c.1026+32T>G |  |  |
| 01-042 | PTEN-ASD | c.1003C>T | p.Arg335* | Truncation |  |  |  |
| 02-001 | PTEN-ASD | c.389G>A | p.Arg130Gln | Missense |  | -2.040 | 0.842 |
| 02-005 | PTEN-ASD | c.728delT | p.Phe243fs*13 | Truncation | c.-1084C>T, c.1026+32T>G |  |  |
| 03-007 | PTEN-ASD | c.165-2A>G | Intron 2, p.Arg55Ser+p.Phe56_Leu70del | Splice/Truncation | c.1026+32T>G |  |  |
| 04-001 | PTEN-ASD | c.683delA | p.Lys228fs*23 | Truncation |  |  |  |
| 04-002 | PTEN-ASD | c.395G>A | p.Gly132Asp | Missense |  | -3.362 | 0.444 |
| 04-003 | PTEN-ASD | c.388C>T | p.Arg130* | Truncation | c.1026+32T>G |  |  |
| 04-005 | PTEN-ASD | c.-729C>T | (promotor) | Haploinsufficiency (Decreased Expression) | c.1026+32T>G |  |  |
| 04-011 | PTEN-ASD | c.389G>A | p.Arg130Gln | Missense | c.1026+32T>G | -2.040 | 0.842 |
| 04-012 | PTEN-ASD | c.999dupC | p.Asn334GInfs*9 | Truncation |  |  |  |

**Note.** All *PTEN* patients had macrocephaly defined as >97^th^ percentile per age norms. Fitness and abundance scores are only valid for missense mutations.

**Supplemental Table S3**. Neurobehavioral measures across study groups.

|  | **(1) Macro-ASD** | **(2) PTEN no-ASD** | **(3) PTEN-ASD** | **F (p)** | **1 vs. 2** | **1 vs. 3** | **2 vs. 3** |
| --- | --- | --- | --- | --- | --- | --- | --- |
|  | M (SD) | M (SD) | M (SD) |  | d | d | d |
| Full Scale IQ | 68.3 (24.3) | 101.0 (23.2) | 55.8 (22.5) | 18.3 (<.001) | *-1.38* | .53 | *1.98* |
| Verbal IQ | 68.1 (24.8) | 101.7 (24.0) | 57.0 (23.0) | 16.1 (<.001) | *-1.38* | .46 | *1.90* |
| Non-verbal IQ | 71.4 (23.8) | 102.2 (23.6) | 57.2 (23.0) | 20.1 (<.001) | *-1.30* | .61 | *1.93* |
| Language composite (z) | -1.9 (1.9) | 0.4 (1.8) | -2.9 (1.8) | 16.5 (<.001) | *-1.24* | .54 | *1.83* |
| Frontal-subcortical composite (z) | -1.3 (0.7) | -0.7 (0.7) | -1.9 (0.7) | 16.5 (<.001) | *-.86* | *.86* | *1.71* |
| Attention composite (z) | -1.1 (0.8) | -0.8 (0.8) | -1.7 (0.8) | 6.5 (.003) | -.38 | *.75* | *1.13* |
| Working memory (z) | -2.0 (1.7) | 0.4 (1.8) | -2.7 (1.6) | 20.6 (<.001) | *-1.37* | .42 | *1.82* |
| Processing speed (z) | -1.9 (1.3) | -0.5 (1.2) | -2.4 (1.2) | 12.0 (<.001) | *-1.12* | .40 | *1.58* |
| Motor skills composite (z) | -1.3 (0.8) | -0.8 (0.8) | -2.0 (0.8) | 11.9 (<.001) | -.63 | *.88* | *1.50* |
| Internalizing problems (T-score) | 61.6 (40.5) | 58.4 (27.2) | 60.4 (23.5) | 0.9 (.914) | .09 | .04 | -.08 |
| Externalizing problems (T-score) | 49.3 (32.4) | 43.7 (27.2) | 52.4 (26.5) | 4.07 (.022) | .19 | -.10 | -.32 |
| Autism traits (SRS-2: T-score) | 73.0 (22.9) | 60.3 (24.0) | 74.9 (24.0) | 3.4 (.040) | .54 | -.08 | -.61 |
| Repetitive Behavior (RBS-R total raw) | 23.2 (18.2) | 10.2 (17.6) | 29.8 (17.2) | 6.3 (.003) | .73 | -.37 | *-1.13* |
| Adaptive Behavior (SS) | 73.6 (23.8) | 97.2 (22.8) | 61.2 (22.5) | 12.8 (<.001) | *-1.01* | .54 | *1.59* |

Note. Means and SDs obtained from pooled estimates. F values obtained from first imputation with age and sex as covariates. IQ scores are provided in standard score units. Behavior problems and autism traits are provided in T-score units. Repetitive behavior is presented in raw score units. All other neurobehavioral scores are provided in z-scores. Italicized Cohen’s d value indicates a significant group difference (p<.05).

**Supplemental Table S4**. Bivariate correlations between PTEN-related proteins and neurobehavioral measures.

|  | PTEN | P-AKT | Total AKT | P-ERK | Total ERK | MnSOD | IGFBP2 | P-S6 | S6 | EIF2A | p27 |
| --- | --- | --- | --- | --- | --- | --- | --- | --- | --- | --- | --- |
| Full Scale IQ | -.26* | .19 | .28* | -.04 | .17 | .04 | .10 | .01 | .17 | .28* | .16 |
| Verbal IQ | -.25^ | .19 | .27* | .01 | .16 | .07 | .09 | -.02 | .16 | .27* | .12 |
| Non-verbal IQ | -.27* | .16 | .26* | -.08 | .17 | -.01 | .09 | .03 | .19 | .28* | .20 |
| Language (z) | -.24^ | .15 | .22^ | -.03 | .18 | .04 | .06 | -.03 | .23^ | .30* | .15 |
| Frontal-subcortical function (z) | -.23^ | .17 | .16 | .05 | .07 | .03 | -.01 | -.01 | .09 | .17 | .29* |
| Attention (z) | -.16 | .16 | .06 | .03 | .02 | .05 | -.08 | .05 | .10 | .15 | .26* |
| Working memory (z) | -.25^ | .15 | .24^ | -.06 | .14 | .05 | .08 | .03 | .09 | .27* | .13 |
| Processing speed (z) | -.18 | .06 | .11 | .11 | -.05 | -.06 | -.05 | -.06 | .09 | .17 | .24^ |
| Motor skills (z) | -.14 | .11 | .18 | .03 | .12 | .01 | .07 | .01 | .08 | .06 | .31* |
| Internalizing problems (T-score) | .03 | .01 | .10 | .07 | -.07 | -.09 | .07 | .03 | .21^ | .07 | -.03 |
| Externalizing problems (T-score) | .09 | -.30* | -.26* | -.13 | -.03 | .11 | -.15 | .19 | -.21 | -.15 | -.12 |
| Autism traits (SRS-2 T-score) | .18 | -.25^ | .02 | -.08 | -.04 | -.15 | .03 | .10 | .14 | -.12 | .06 |
| Repetitive Behavior (RBS-R total) | .08 | -.12 | -.16 | .13 | -.13 | .07 | -.16 | .27* | -.18 | -.08 | -.01 |
| Adaptive Behavior Composite (SS) | -.24^ | .03 | .13 | .02 | .14 | .20 | .10 | .05 | -.01 | .17 | .08 |

Note. N=61, ^p<.10, *p<.05. 15 of 154 correlations have p<.05 two-tailed. The probability of a number of significant tests this high or larger is p=.005. 26 of 154 correlations have p<.10 two-tailed. The probability of a number of significant tests this high or larger is p=.003.

**Supplemental Table S5.** Bivariate correlations between AKT, ERK, and S6 ratios and neurobehavior measures.

|  | P-AKT/AKT | P-ERK/ERK | P-S6/S6 |
| --- | --- | --- | --- |
| Full Scale IQ | -.19 | -.12 | -.07 |
| Verbal IQ | -.15 | -.10 | -.08 |
| Non-verbal IQ | -.21 | -.14 | -.04 |
| Language (z) | -.14 | -.12 | -.13 |
| Frontal-subcortical function (z) | -.08 | -.03 | -.01 |
| Attention (z) | -.01 | .01 | .06 |
| Working memory (z) | -.20 | -.10 | -.01 |
| Processing speed (z) | -.12 | .08 | -.07 |
| Motor skills (z) | -.08 | -.07 | .01 |
| Internalizing problems (T-score) | -.17 | .10 | -.11 |
| Externalizing problems (T-score) | .19 | -.08 | .33* |
| Autism traits (SRS-2 T-score) | -.26* | .01 | .06 |
| Repetitive Behavior (RBS-R total) | .12 | .14 | .38** |
| Adaptive Behavior Composite (SS) | -.06 | -.12 | .07 |

Note. N=61, *p<.05, **p<.001.

**Supplemental Figure S1.** Relationships between p27 and frontal sub-cortical, attentional, and motor function composite scores.


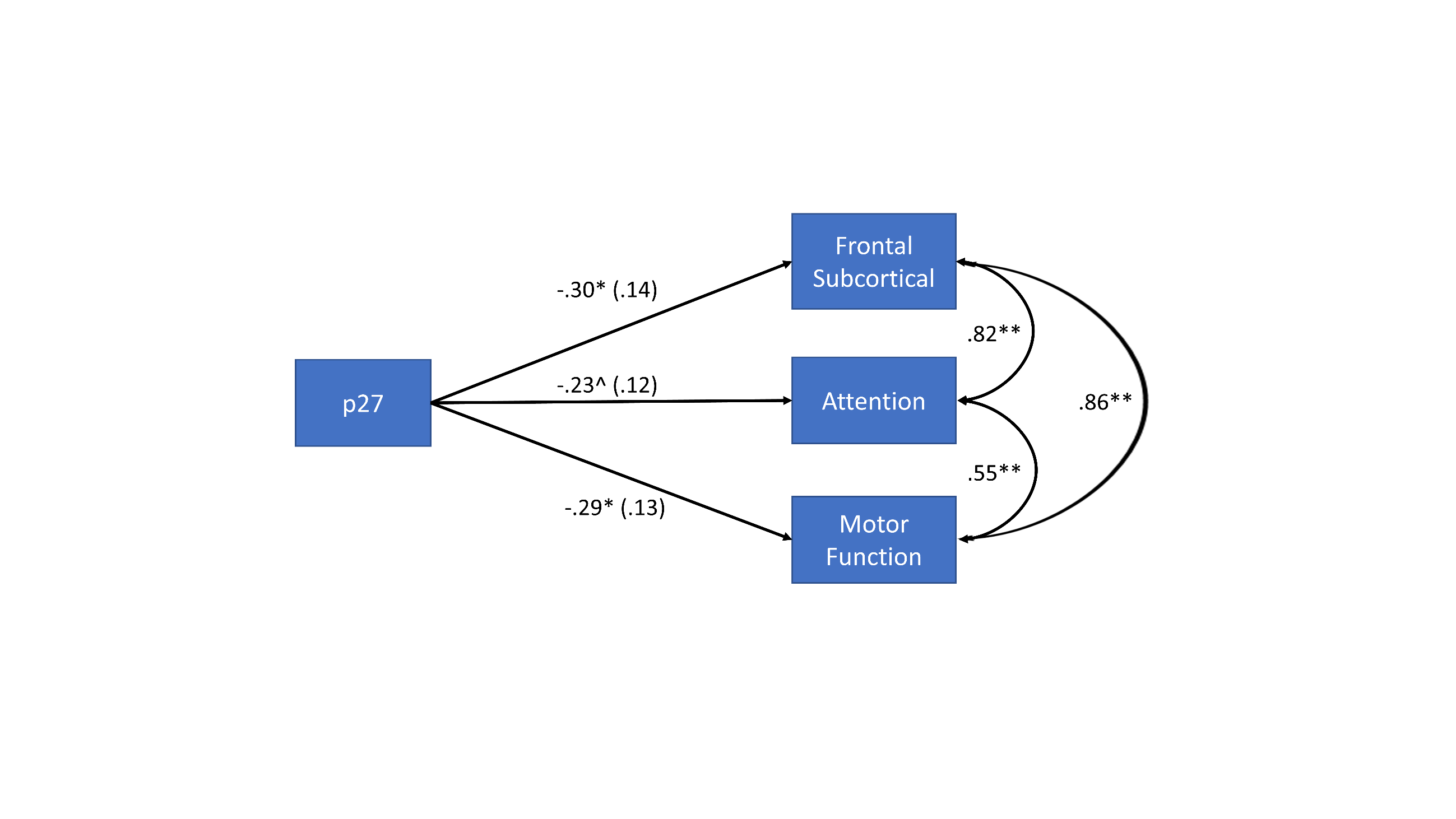


**Note.** ^p<.10, *p<.05, **p<.001.
